# Supplementary material for: Surgical debulking of podoconiosis nodules and its impact on quality of life in Ethiopia
Source: PLoS Negl Trop Dis. 2021 Jan 22;15(1):e0009053. doi: 10.1371/journal.pntd.0009053 (PMC7857577; doi:10.1371/journal.pntd.0009053)
Supplement: Attachment — Submitted filename: Point by point response06112020 HdV.docx [file pntd.0009053.s001.docx]

Point by point response

Reviewer #1
This paper has a very good approach related to the assessment of Dermatology quality of live index in patients with surgical treatment including those with a late stage of severity.
I would like to see the inclusion in the diagnosis section of the BinaxNOW filariasis rapid antigen test to rule out LF in podoconiosis, where doubt remains (WHO 2011). Please clarify

A: Thank you for the constructive comment. The diagnosis of podoconiosis was only clinical (based on the criteria I have stated in the methodology) and no blood sample was collected to perform antigen test. And I will address your comment on the limitation of the study (line 256)

Reviewer #2
Authors describe a descriptive analysis of outcomes of nodulectomy in patients with chronic podoconiosis. The results are encouraging and it would prove to be of a high clinical relevance. Few minor corrections can enhance the paper:

*Please recheck your manuscripts throughout for typos and grammar issues. Please use American and not British English (e.g. edema and not oedema).

A: Thanks I did correct that
*Also in several passages through the text there is a lack of an appropriate space among words including the abstract (see few examples in lines 16, 97, 127, 135, 136, 152, 168, etc.) Please check the entire manuscript carefully.

A: Thanks I did corrected the spacing.
Methods:
*Please clarify in the method and results section, what other additional interventions were offered to those patients that could have impacted the DLQI scores after surgery? were the patients provided with shoes? Were they given pain or other medications, access to healthcare?

A: Thank you, one important thing patients were provided with was a lymphedema morbidity management service (patient was advised on daily foot wash, use of moisturizer and for those with water bag lymphedema short stretched elastic bandage was given). The antibiotic and analgesic use is mentioned in the methodology and result part. (I have further clarified it in the methods) line 136-138

* How this group of patients compared to the most general population? How was your patients selected to the study?

A: As documented, we included patients who have presented seeking medical service for lymphedema and nodules. A description of the general population is presented in the lines 120-126.

Discussion:
* Please discuss more limitations of your study. What other unforeseen interventions/biases could have affected the results?

A: Thanks, I have added the additional interventions, line 257

*Please expand and theorize why the intervention worked? Better functionality, less stigma, less progression?

A: Thank you, I have addressed that in the discussion.
*Please describe and compare your study findings with the natural one year progression of the disease without intervention. How is it different?

A: Patient with non-pitting edema and woody-hard fibrous nodules, they tend to have difficulty to get shoes for their foot, have recurrent disabling bacterial infection and unresponsive to the above mentioned therapy which makes nodulectomy the last resort management for those patients. (I have added a sentence in the manuscript )

*Please propose a follow up study to confirm the findings of this preliminary/descriptive study

A: Thank you and I have added a sentence to address that ‘We recommend a future case control studies in different centres to validate the current result and address some of the limitation of this study.’
